# Methyltransferase-Like 3-Mediated m6A Methylation of Hsa_circ_0058493 Accelerates Hepatocellular Carcinoma Progression by Binding to YTH Domain-Containing Protein 1

**DOI:** 10.3389/fcell.2021.762588

**Published:** 2021-11-23

**Authors:** Anqi Wu, Yuhao Hu, Yao Xu, Jing Xu, Xinyue Wang, Aiting Cai, Ruoyu Liu, Lin Chen, Feng Wang

**Affiliations:** ^1^Department of Laboratory Medicine, Affiliated Hospital of Nantong University, Nantong, China; ^2^Department of Laboratory Medicine, The Second Affiliated Hospital of Nantong University, Nantong, China; ^3^Department of Laboratory Medicine, School of Public Health, Nantong University, Nantong, China; ^4^Department of Hepatology Laboratory, Nantong Third Hospital Affiliated to Nantong University, Nantong, China

**Keywords:** circular RNA, HCC, N6-methyladenosine, hsa_circ_0058493, METTL3, YTHDC1

## Abstract

Circular RNAs (circRNAs) are highly correlated with the progression and prognosis of hepatocellular carcinoma (HCC). In addition, mounting evidence has revealed that N6-methyladenosine (m6A) methylation, a common RNA modification, is involved in the progression of malignancies. In this research, a novel circRNA, hsa_circ_0058493, was proven to be upregulated in HCC, which was correlated with the prognosis of HCC patients. Experimentally, hsa_circ_0058493 knockdown suppressed the growth and metastasis of HCC cells *in vivo* and *in vitro*. On the contrary, the overexpression of hsa_circ_0058493 in HCC cells had the opposite effect *in vitro*. Mechanistic experiments revealed that hsa_circ_0058493 contained m6A methylation sites and that methyltransferase-like 3 (METTL3) mediated the degree of methylation modification of hsa_circ_0058493. Furthermore, YTH domain-containing protein 1 (YTHDC1) could bind to hsa_circ_0058493 and promote its intracellular localization from the nucleus to the cytoplasm. In addition, both si-METTL3 and si-YTHDC1 suppressed HCC cell growth and metastasis, whereas rescue experiments confirmed that overexpression of hsa_circ_0058493 inverted the inhibitory effects of si-METTL3 and si-YTHDC1 on HCC cells. Taken together, this study explored the oncogenic role of m6A-modified hsa_circ_0058493 and found to accelerate HCC progression via the METTL3-hsa_circ_0058493-YTHDC1 axis, indicating a potential therapeutic target for this deadly disease.

## Introduction

Hepatocellular carcinoma (HCC), one of malignant tumors in the world with high incidence and mortality (Chen et al., 2019c; Lan et al., 2019). In addition, the high postoperative recurrence rate and metastasis rate lead to the poor prognosis of HCC patients (Zhang and Zhang, 2019; Wang et al., 2020d). Because of the scarcity of effective treatments, patients are often diagnosed with advanced HCC without receiving timely treatment (Zhang et al., 2018; Zhao et al., 2020). In view of the above, the clinical biomarkers used to diagnose HCC are not specific (Liu et al., 2021). To clarify the mechanism of the occurrence and development of HCC, it is necessary to seek biomarkers for the early diagnosis of HCC (Xu et al., 2020).

Circular RNAs (circRNAs) are a class of non-coding RNAs which have covalently closed circular structure. Furthermore, circular ribonucleic acid, without a 5′ end and 3′ tail structure, is more stable than the corresponding linear ribonucleic acid (Geng et al., 2018; Han et al., 2021). A growing number of studies have elucidated the mechanism of circRNAs in many malignant tumors (Latowska et al., 2020). For example, a potential diagnostic biomarker, circTMEM45A, may promote HCC progression through the miR-665/IGF2 axis by acting as a sponge for microRNA-665 (Zhang et al., 2020). In addition, circ-FBXW7 is an endogenous circRNA with translational function, and circ-FBXW7 and its encoded protein FBXW7-185aa have a certain prognostic value for glioblastoma (Yang et al., 2018). It is commonly known that circRNAs have high stability and can be used as new biomarkers for disease diagnosis or prognosis (Lei et al., 2020). However, the potential function of cyclic ribonucleic acid in HCC is still under investigation.

As a main modification in eukaryotic mRNA, N6-methyladenosine (m6A) plays vital roles in cancers (Li and Zhan, 2020; Chen et al., 2021). Many reports have proven that there are three players in m6A modification. These are the so-called m6A-related “writers,” “erasers,” and “readers” which mediate the methylation process, participate in the demethylation process and participate in RNA recognition, respectively (Qian et al., 2019; Pan et al., 2020). Methyltransferase-like 3 and 14 proteins (METTL3 and METTL14) are the “writers” of m6A. The relevant literature has reported that METTL3 is overexpressed in HCC, while METTL14 is underexpressed in HCC (Liu et al., 2020). Moreover, the YT521-B homology (YTH) domain family protein is a characteristic m6A reader (Xu et al., 2015). Research has shown that YTH domain-containing protein 1 (YTHDC1) promotes the nuclear export of circNSUN2 which bind to m6A motifs, ultimately promoting colorectal cancer progression (Chen et al., 2019a). Moreover, m6A modification also influences mRNA translation, splicing, export, degradation and processing (Wang et al., 2020a). However, the relationship between m6A modification and circRNAs in HCC needs to be further explored.

In the current research, we concluded that hsa_circ_0058493 was upregulated in HCC. Moreover, we have studied that hsa_circ_0058493 was regulated by m6A methylation and promoted the progression of HCC by binding to YTHDC1. In summary, hsa_circ_0058493 is expected to be a therapeutic target for HCC.

## Materials and Methods

### Patient Tissue Specimens

Fifty-one pairs of HCC tissue and paired normal tissue were gathered from the Affiliated Hospital of Nantong University from January 2015 to December 2016. The clinical data of all patients was used with their informed consents. The Ethics Committee of the Affiliated Hospital of Nantong University authorized the agreement of the organization used for this study.

### Cell Culture and Transfection

In this study, HCC cell lines (BEL-7404, HCCLM3, SK-Hep-1, SMMC-7721, and MHCC-97H) together with a normal liver cell line (LO2) were purchased from the Chinese Academy of Sciences. All cells were cultured in high glucose Dulbecco’s Modified Eagle’s Medium (Corning, NY, United States), which contained 10% fetal bovine serum (FBS, Gibco, Grand Island, NY, United States) and 1% penicillin-streptomycin-amphotericin B solution (Solarbio, Beijing, China), at 37°C with 5% CO_2_ in an incubator. HCCLM3 and SMMC-7721 cells were treated with negative control (shNC) and sh-hsa_circ_0058493 (sh-circ-1, sh-circ-2) or negative control (oe-NC) and oe-hsa_circ_0058493 (oe-circ). The transfection plasmid was provided by Geneseed Biotech Co., Ltd. Cells were cultured for 48 h before transfection. When the cells have grown to 70–80% density of the six-well plate, we transfected the cells with Lipofectamine 3000. After 48 h of transfection, RT–qPCR assays were used to detect the transfection efficiency and carry out follow-up experiments. The sequences of the negative control (shNC) were TCACCAGAAGCGTACCATACTC, and the sequences of sh-hsa_circ_0058493 were ATACAGACGGCT GAACCCTGGTGAG (sh-circ-1) and ACAGACGGCTGAAC CCTGGTGAGAA (sh-circ-2). The sequences of si-METTL3 were GCACTTGGATCTACGGAA and the sequences of si-YTHDC1 were CAAGGAGTGTTATCTTAAT.

### Animal Studies

All experiments were approved by the Institutional Animal Care and Utilization Committee of Nantong University. sh-hsa_circ_0058493 and its negative control cells were stably transfected into HCCLM3 cells. Approximately 1 × 10^7^ cells were injected subcutaneously into the armpit of nude male mice (4 weeks old, 10 in total, divided into five mice per group). The growth of the tumor was recorded by measuring the size with a caliper every week. After 4–5 weeks, the tumors were removed from the mice and their volume and weight were recorded. Besides, the tumors were made into paraffin-embedded sections for HE staining and immunohistochemical examination (IHC).

### Immunohistochemical Staining

After fixing the tumor with 4% paraformaldehyde, paraffin-embedded sections were prepared. After sectioning the tumor, the slices were degreased in xylene and then subjected to microwave heating treatment to extract the antigen. Next, the sections were incubated with PCNA, Ki67 and Bcl-2 and secondary antibody (Santa Cruz, CA, United States). After being washed, the sections were stained with hematoxylin and 3,3′-diaminobenzidine (DAB). Finally, the slices were imaged and observed with an inverted microscope.

### RNA Extraction, Reverse Transcription, and RT–qPCR

Total RNA was isolated by using TRIzol reagent (Invitrogen). Then, a reverse transcription kit was used to convert total RNA into cDNA (Thermo Fisher Science, United States). The expression levels of circRNAs and mRNAs were amplified on a LightCycler 480 qRT-PCR instrument (Roche, Germany) with Plus SYBR real-time PCR mixture (BioTeke, Beijing, China). Samples were subjected to reaction conditions of 15 s at 95°C, 30 s at 60°C, 30 s at 75°C, and 45 cycles. Each sample was repeated three times. The comparative cycle threshold values (2^–ΔΔCt^) were calculated to analyze the expression level of circRNAs and mRNAs. The primer information is shown in Table 1.

**TABLE 1:** Sequence information for primers used in this study.

**Gene**	**Sequence (5′-3′)**
hsa_circ_0058493 (divergent primers)	F: TATCTGGCCATGCAACGGAG
	R: TCACCCTAGCAACTTTGGCC
hsa_circ_0058493 (convergent primers)	F: ATTCTCACCAGGGTTCAGCC
	R: CTCCGTTGCATGGCCAGATA
METTL3	F: CTTCAGTTCCTGAATTAGC
	R: ATGTTAAGGCCAGATCAGAGAG
YTHDC1	F: ATCTTCCGTTCGTGCTGT
	R: ACCATACACCCTTCGCTTT
18s	F: CGGCTACCACATCCAAGGAA
	R: GCTGGAATTACCGCGGCT

### Cell Proliferation and Clone Formation Assay

Cell proliferation ability was evaluated by a CCK-8 kit (MedChemExpress, Shanghai, China). The transfected cells were developed in 96-well plate at a density of 3,000 cells/well. At 24, 48, 72, 96, and 120 h after inoculation, after adding 10 μl of CCK-8 solution to each well, absorbance value was measured at 450 nm after 2 h of incubation. In the colony formation assay, approximately 1,000 cells per well of transfected cells were added into a six-well plate with 2 weeks’ incubation. Next, the cells were fixed with 4% formaldehyde and stained with 0.1% crystal violet. Finally, clonal spots were photographed and counted.

### Cell Migration and Invasion Assays

In cell migration experiment, 5 × 10^5^/ml transfected cells were seeded into the upper chambers with DMEM without serum. In addition, the lower chambers were added with DMEM containing 10% FBS. Similarly, in cell invasion experiment, 7 × 10^5^/ml transfected cells were cultured in chambers covered with 100 μl Matrigel (1:10 dilution; BD Biosciences). After incubation for 48 h, the chambers were fixed and stained as above. After wiping the upper chamber with a cotton swab, we used a microscope to count the number of migrated and invaded cells at the bottom of the chambers.

### Cell Cycle Assay and Apoptosis Experiments

The treated cells were all collected and fixed with 70% ethanol. After removing the ethanol, the cells were washed three times with phosphate buffer. Fifty microliters of enzyme was added to each tube, which were incubated in a bath at 37°C. Then, 200 μl dye was added to each tube in the dark and incubated on ice. After the transfected cells were cultured, the cells and the dead cells in the six-well plate were collected. Cells were resuspended in PBS for collection and stained with Annexin V-Alexa Fluor 647/PI. Cell cycle assays and apoptosis experiments were performed by flow cytometry (BD Bioscience, United States). The results were statistically analyzed.

### RNA Immunoprecipitation Assay

According to the instructions, the RIP experiment was performed with an RNA binding protein immunoprecipitation kit (Geneseed Biotech, Guangzhou, China). A total of 1 × 10^7^ cells were added to the lysate, and 100 μl of the supernatant was used as a positive control. The magnetic beads were coated with 5 μg of anti-YTHDC1 (77422, Cell Signaling Technology), anti-m6A (56593, Cell Signaling Technology), and IgG (2729, Cell Signaling Technology) at 4°C for 2 h. The antibody surface-coated magnetic beads and cell lysate were incubated overnight at 4°C, and the magnetic bead-protein-RNA complex was washed with RIP washing buffer. Cell lysate was added to the magnetic bead complex-antibody to capture the antigen. After eluting the complex bound to the magnetic beads, the RNA was extracted with a filter column. The expression of hsa_circ_0058493 was determined by an RT–qPCR assay.

### Nuclear and Cytoplasmic Extraction

Nuclear and cytoplasmic RNA were separated and extracted by nuclear and cytoplasmic protein extraction kits (Beyotime Biotechnology). Then, the expression of hsa_circ_0058493 in the cytoplasm and nucleus was analyzed by RT–qPCR assay.

### Statistical Analysis

The data were analyzed with SPSS 21.0 statistical software. A *P* < 0.05 was regarded as statistically significant for two-sided analysis. All data was presented as the mean ± standard deviation. Comparisons between groups were analyzed by *t*-test or ANOVA.

## Results

### Characterization and Detection of Hsa_circ_0058493 in Hepatocellular Carcinoma

**FIGURE 1:**
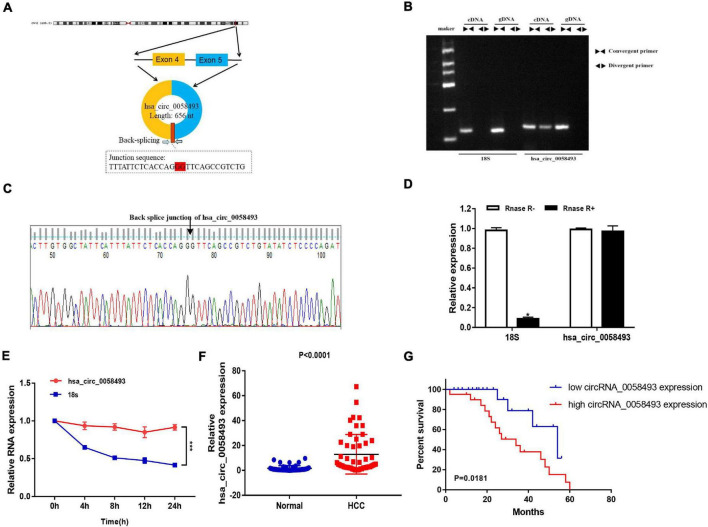
**(A)** The circular structure of hsa_circ_0058493 showed that the hsa_circ_0058493 primer was derived from exons 4–5 of the RHBDD1 gene. Yellow represents exon4, blue represents exon5, and red represents the Back-splicing. **(B)** Gel electrophoresis results showed that the head-to-head primers can amplify products in both gDNA and cDNA, while the back-to-back primers can only amplify hsa_circ_0058493 in cDNA, which proved that hsa_circ_0058493 had a circular structure. **(C)** The reverse splicing junction of hsa_circ_0058493 was detected by Sanger sequencing. **(D)** The results of the RNase R enzyme digestion test showed that the expression of linear RNA decreased visibly after RNase R enzyme digestion, while the expression of hsa_circ_0058493 after RNase R enzyme treatment did not change clearly. **(E)** After treatment with actinomycin D in HCCLM3 cells, the relative RNA expression of hsa_circ_0058493 and mRNA were detected at different times. **(F)** Relative expression of hsa_circ_0058493 in 51 pairs of HCC and normal tissues. **(G)** The overall survival curve with low and high expression of hsa_circ_0058493 in HCC was analyzed by Kaplan–Meier. *P < 0.05, ***P < 0.001 vs. control group.

We screened and detected the expression of 5 circRNAs in HCC from the GSE97332 database and GSE97508 database. Hsa_circ_0058493 expression in HCC tissues was distinctly increased, and we studied the circular structure of hsa_circ_0058493 and discovered hsa_circ_0058493 was derived from exons 4–5 of the RHBDD1 gene (Figure 1A). To characterize hsa_circ_0058493, we designed convergent primers and divergent primers. Also, agarose gel electrophoresis results showed that hsa_circ_0058493 was amplified from complementary DNA (cDNA) instead of genomic DNA (gDNA) (Figure 1B). Furthermore, Sanger sequencing confirmed that there was a back-splicing junction (Figure 1C). Additionally, RNase R specifically degrades linear RNAs rather than circRNAs, and we confirmed that hsa_circ_0058493 can resist the digestion of RNase R (Figure 1D). After treatment with actinomycin D, qRT-PCR results proved that hsa_circ_0058493 possessed a longer half-life than the mRNA. Hsa_circ_0058493 was more stable than mRNA due to its ring structure (Figure 1E). Next, we studied the expression profile of hsa_circ_0058493 in 51 pairs of tissues. The expression of hsa_circ_0058493 in HCC tissues was memorably upregulated (Figure 1F). Kaplan Meier analysis showed that HCC patients in hsa_circ_0058493 high expression group had a worse prognosis and shorter survival time (Figure 1G). In summary, these results indicated that hsa_circ_0058493 has a true ring structure and is generally upregulated in HCC tissues. Additionally, hsa_circ_0058493 was connected with the progression of HCC and may become a promising prognostic marker for HCC.

### Hsa_circ_0058493 Promoted the Growth and Metastasis of Hepatocellular Carcinoma Cells

**FIGURE 2:**
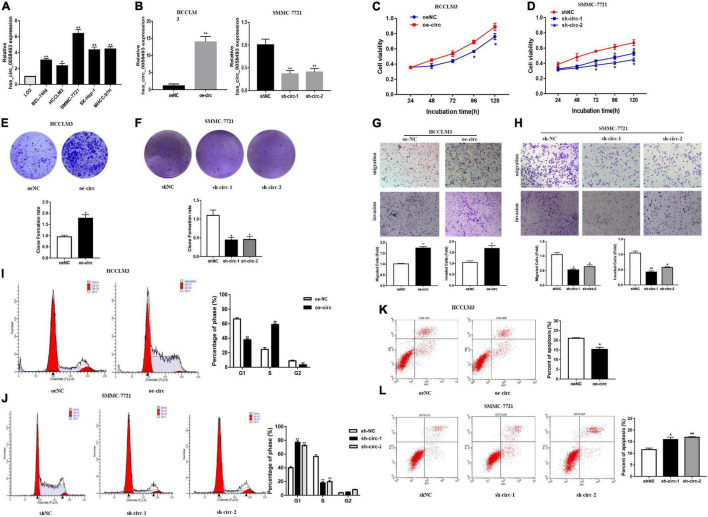
**(A)** The expression of hsa_circ_0058493 in BEL-7404, HCCLM3, SMMC-7721, SK-Hep-1, MHCC-97H, and LO2 cells was detected by qRT–PCR assays. **(B)** The efficiency of transfecting the knockout plasmid and overexpression plasmid of hsa_circ_0058493 into HCC cells by qRT-PCR. **(C,D)** The CCK-8 test was used to analyze the proliferation of HCC cells with overexpression and knockdown of hsa_circ_0058493 (oe-circ and sh-circ-1, sh-circ-2). **(E,F)** Cloning experiments were tested to analyze the effect of oe-circ and sh-circ-1, sh-circ-2 on the proliferation of HCC cells. The colony formation rate was shown by a histogram. **(G,H)** The migration and invasion ability of HCC cells with oe-circ and sh-circ-1, sh-circ-2 was tested by the Transwell assay. The number of migrating and invading cells was counted. **(I,J)** Cell cycle assays were analyzed by flow cytometry. The histogram showed that hsa_circ_0058493 knockdown cells stagnated in G1 phase and that hsa_circ_0058493-overexpressing cells promoted cell proliferation. The triangle symbols are for discrimination of G1 vs. S and S vs. G2. **(K,L)** Apoptosis was detected by flow cytometry. The assay proved that the number of apoptotic cells detected in the samples transfected with the overexpression of hsa_circ_0058493 (oe-circ) was small, while the samples transfected with knockdown of hsa_circ_0058493 (sh-circ-1, sh-circ-2) had more apoptotic cells. *P < 0.05, **P < 0.01 vs. control group.

In order to discover the role of hsa_circ_0058493 in HCC cells, we first performed qRT-PCR assays to detect the expression of hsa_circ_0058493 in HCC cell lines (Figure 2A). Then, knockdown and overexpression plasmids were used to stably transfect HCC cell lines HCCLM3 and SMMC-7721, respectively, and a negative control was used (Figure 2B). The CCK-8 test results displayed that overexpression of hsa_circ_0058493 (oe-circ) facilitated cell proliferation, alternatively knockdown of hsa_circ_0058493 (sh-circ-1, sh-circ-2) inhibited cell proliferation (Figures 2C,D). The clone formation experiment exhibited that compared with the negative control, the oe-circ raised the number of clones of HCCLM3 cells, nevertheless, sh-circ reduced the number of clones of SMMC-7721 cells (Figures 2E,F). The above results proved the proliferation ability of hsa_circ_0058493 in HCC. Furthermore, the Transwell assay confirmed that the migration and invasion ability of oe-circ cells was better than that of the negative control (oe-NC), whereas, the migration and invasion ability of sh-circ cells was worse than that of the negative control (sh-NC) (Figures 2G,H). Afterward, flow cytometry analysis showed that the number of oe-circ cells in S phase increased, while the percentage of sh-circ cells in G1 phase increased. The results proved that oe-circ in HCC cells rescued cell cycle arrest and promoted cell proliferation (Figures 2I,J). Moreover, cell apoptosis was detected by Annexin V and PI double staining kits. The results indicated that oe-circ subtracted apoptotic cells, while sh-circ increased the number of apoptotic cells (Figures 2K,L).

### Downregulation of Hsa_circ_0058493 Inhibited the Growth of Hepatocellular Carcinoma Tumors *in vivo*

**FIGURE 3:**
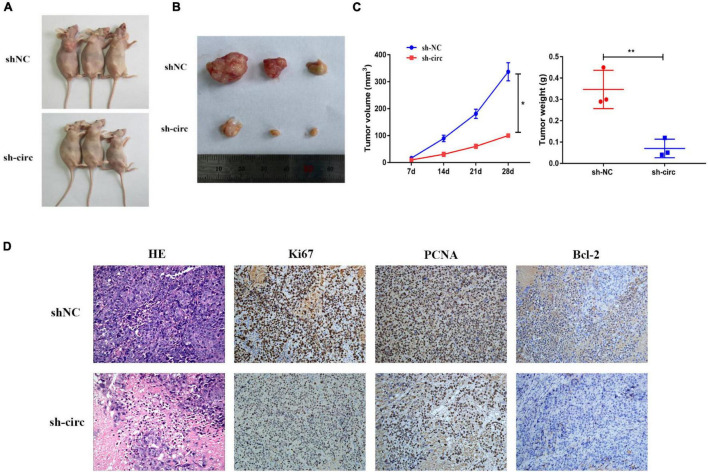
**(A)** Image of nude mice 28 days after inoculation with sh-NC (control) and sh-circ plasmids. **(B)** Representative images of subcutaneous tumors in nude mice. **(C)** The tumor growth volume and weight measurements of nude mice showed that the growth of tumors was inhibited after hsa_circ_0058493 was knocked down. **(D)** The expression of Ki67, PCNA, and Bcl-2 (tumor proliferation marker and tumor apoptosis marker) in nude mice were detected by H&E staining and IHC staining. *P < 0.05, **P < 0.01 vs. control group.

In nude mouse subcutaneous tumor formation experiments, we stably transfected hsa_circ_0058493 knockdown cells into the HCCLM3 cell line and inoculated the HCCLM3 cell line into the skin of nude mice. Then, we observed the growth of subcutaneous tumors every week. Silencing of hsa_circ_0058493 significantly inhibited the growth of subcutaneous tumors (Figures 3A,B). In addition, the tumors injected with the transfected knockdown plasmid grew much slower than those injected with the negative control plasmid. Compared with sh-NC group, the tumor volume and weight of the sh-circ group were notably reduced (Figure 3C). Besides, the results of H&E and immunohistochemistry staining showed that in hsa_circ_0058493 knockdown group, the positive rate of Ki67, PCNA (tumor proliferation marker) and Bcl-2 (tumor apoptosis marker) *in vivo* was markedly reduced compared with sh-NC (Figure 3D). These experimental data indicated that hsa_circ_0058493 promoted the growth of HCC tumors *in vivo*.

### Methyltransferase-Like 3 Catalyzed the m6A Modification of Hsa_circ_0058493

**FIGURE 4:**
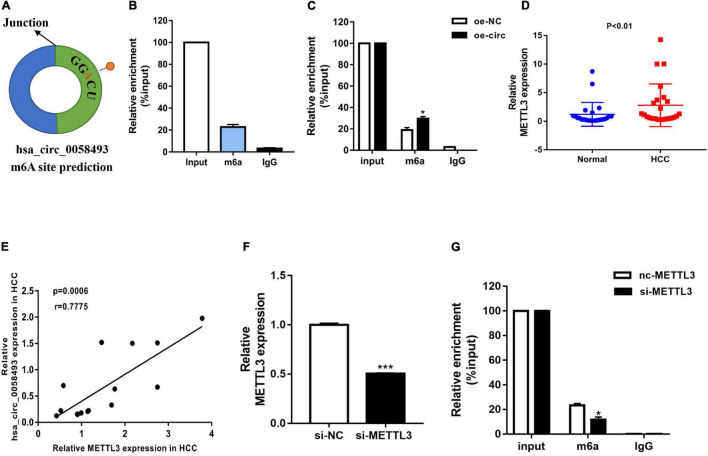
**(A)** The SRAMP database predicted the GGACU m6A site in hsa_circ_0058493. **(B)** MeRIP experiment shows that circNSUN2 is relatively enriched in m6A antibody compared with control IgG antibody. **(C)** When the hsa_circ_0058493 overexpression plasmid was transfected, the MeRIP experiment proved that the m6A antibody was more enriched. **(D)** Relative expression of METTL3 in 12 pairs of HCC and adjacent tissues was detected by qRT-PCR. **(E)** Hsa_circ_0058493 was positively correlated with METTL3. The *P* value and correlation coefficient were calculated by Pearson correlation analysis. **(F)** The knockdown efficiency of the METTL3 knockdown plasmid was tested by qRT-PCR. **(G)** MeRIP experiment proved that after knocking down METTL3 in hsa_circ_0058493, the degree of m6A methylation decreased. *P < 0.05, ***P < 0.001 vs. control group.

N6-methyladenosine is considered to be a common mRNA modification. It is known that circRNAs containing “RRm6ACH” (R = G or A, H = A, C or U) are more prone to m6A modification. We used the SRAMP database to predict the m6A site, and we found that it was close to the junction site of hsa_circ_0058493 (Figure 4A). To explore whether hsa_circ_0058493 contained m6A methylation, we performed a methylated RNA immunoprecipitation (MeRIP) test. Compared with the control IgG, the complex precipitated by the anti-m6A antibody was enriched in hsa_circ_0058493 (Figure 4B). Furthermore, the RNA-binding protein immunoprecipitation (RIP) assay proved that compared with oe-NC, the degree of methylation of hsa_circ_0058493 transfected with the oe-circ plasmid was increased (Figure 4C). Methyltransferase-like 3 (METTL3) is called an N6-methyladenosine “writer” and plays a vital role in catalyzing m6A modification. Studies have shown that METTL3 is upregulated in HCC. qRT-PCR experiments were used to verify that METTL3 was upregulated in HCC tissues (Figure 4D). Additionally, the qRT-PCR experimental data stated that the expression of METTL3 was positively relevant to hsa_circ_0058493 in HCC tissues (Figure 4E). Next, we constructed a METTL3 knockdown plasmid and performed a series of recovery experiments. The knockdown efficiency of the METTL3 plasmid reached more than 50% (Figure 4F). Subsequently, it was demonstrated by MeRIP that the methylation degree decreased in hsa_circ_0058493 after transfection of si-METTL3 compared with the control group si-NC (Figure 4G). Overall, hsa_circ_0058493 contains methylation sites, and METTL3 promotes the extent of m6A modification in hsa_circ_0058493.

### Methyltransferase-Like 3 Affected the Biological Activity of Hsa_circ_0058493 in Hepatocellular Carcinoma

**FIGURE 5:**
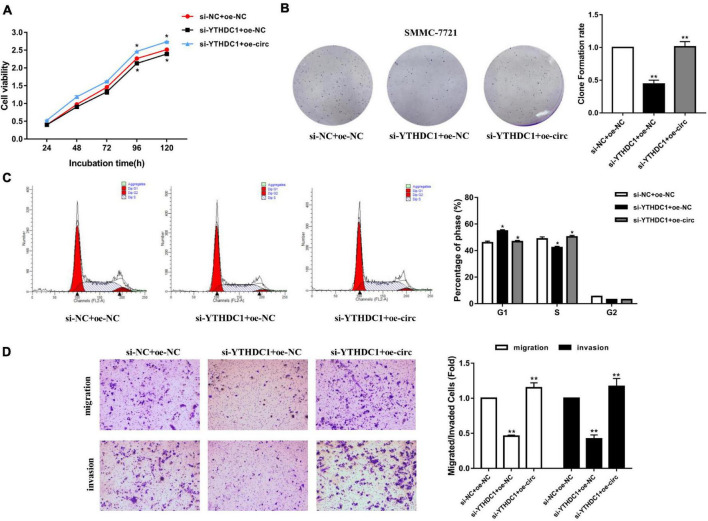
**(A)** A CCK-8 assay was used to determine the proliferation of HCC cells after transfection with si-NC + oe-NC, si-YTHDC1 + oe-NC, and si-YTHDC1 + oe-circ. **(B)** A cloning assay was performed to detected proliferation ability of HCC cells with si-NC + oe-NC, si-YTHDC1 + oe-NC, and si-YTHDC1 + oe-circ. Colony formation rates are shown by histograms. **(C)** The cell cycle of HCC cells after transfection with si-NC + oe-NC, si-YTHDC1 + oe-NC, and si-YTHDC1 + oe-circ was determined by flow cytometry. As evident in the histogram, more cells arrested in the G1 phase were observed in si-YTHDC1 + oe-NC compared with si-NC + oe-NC. After cotransfection of si-YTHDC1 + oe-circ, the number of cells arrested in G1 phase decreased again compared with si-YTHDC1 + oe-NC. The triangle symbols are for discrimination of G1 vs. S and S vs. G2. **(D)** The invasion and migration abilities of HCC cells after transfection with si-NC + oe-NC, si-YTHDC1 + oe-NC, and si-YTHDC1 + oe-circ were assessed by Transwell assay. Histograms were used to show the number of invaded and migrated cells. *P < 0.05, **P < 0.01 vs. control group.

After knocking down METTL3 (si-METTL3) and overexpressing hsa_circ_0058493 (oe-circ) in the SMMC-7721 cell line, the cell proliferation, migration and invasion abilities were tested. The CCK-8 and the clone formation experiments proved that after knocking down METTL3, the cell proliferation ability decreased parallel with control. However, the proliferation ability was rescued after overexpression of hsa_circ_0058493 (Figures 5A,B). Afterward, flow cytometry analysis demonstrated that cell cycle arrest increased and cell growth ability decreased after transfection of the METTL3 knockdown plasmid (si-METTL3) in the SMMC-7721 cell line, but transfection of the hsa_circ_0058493 overexpression plasmid (oe-circ) rescued cell cycle arrest and promoted cell growth ability (Figure 5C). Similarly, the Transwell assay confirmed that after the METTL3 knockdown plasmid (si-METTL3) was transfected into the SMMC-7721 cell line, the cell migration and invasion ability decreased, but after transfection with the hsa_circ_0058493 overexpression plasmid (oe-circ), the cell migration and invasion ability was restored (Figure 5D). Based on the above experimental results, we concluded that METTL3 can affect the extent of m6A modification of hsa_circ_0058493, which in turn affects the growth and metastasis of HCC in response to hsa_circ_0058493.

### YTH Domain-Containing Protein 1 Interacted With Hsa_circ_0058493 and Promoted Cytoplasmic Export of Hsa_circ_0058493

**FIGURE 6:**
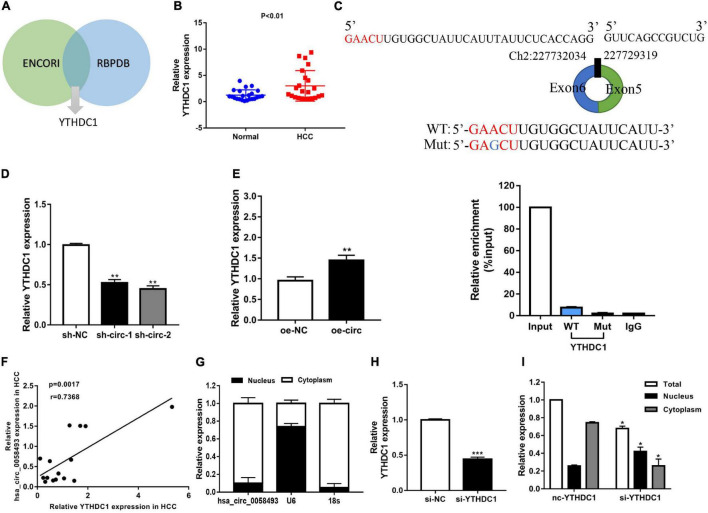
**(A)** Through the intersection of the ENCORI and RBPDB databases, it was concluded that hsa_circ_0058493 may interact with YTHDC1. **(B)** Relative expression of YTHDC1 in 12 pairs of HCC and adjacent tissues was detected by qRT-PCR assays. **(C)** Top, a point mutation in the binding sequence of hsa_circ_0058493 and YTHDC1. Bottom, the RIP experiment proved that YTHDC1 interacted with hsa_circ_0058493 and IgG antibody was used as a control. **(D,E)** The expression of YTHDC1 mRNA was detected by qRT-PCR in cells with hsa_circ_0058493 knockdown plasmid or hsa_circ_0058493 overexpression plasmid. **(F)** Hsa_circ_0058493 was positively correlated with YTHDC1. The *P* value and correlation coefficient were calculated by Pearson correlation analysis. **(G)** Nuclear and cytoplasmic separation experiments showed that hsa_circ_0058493 was in the cytoplasm. U6 was used as a positive control in the nucleus while 18S was used as a positive control in the cytoplasm. **(H)** The knockdown efficiency of the YTHDC1 knockdown plasmid was tested by qRT-PCR. **(I)** The nucleoplasmic separation experiment showed that after knocking down YTHDC1, hsa_circ_0058493 was transported from the cytoplasm to the nucleus. *P < 0.05, **P < 0.01, ***P < 0.001 vs. control group.

Several researches have indicated that circular RNAs can perform a regulatory function by binding related proteins and have a carcinogenic effect in many cancers. We used the ENCORI and RBPDB databases for bioinformatics analysis and found that YTHDC1 may be a binding protein of hsa_circ_0058493 (Figure 6A). The qRT-PCR experiments validated that YTHDC1 expression was upregulated in HCC tissues (Figure 6B). We designed a point mutation in the binding sequence of hsa_circ_0058493 and YTHDC1, and performed the RIP experiment with YTHDC1 antibody. Compared with the negative control IgG, we observed an obvious enrichment of hsa_circ_0058493 in the wild type (WT) group and there was almost not any enrichment rate in the mutant (Mut) group, which verified that YTHDC1 could bind to hsa_circ_0058493 at the prediction site (Figure 6C). We found that YTHDC1 mRNA expression decreased when hsa_circ_0058493 was knocked down, while the expression increased when hsa_circ_0058493 was overexpressed (Figures 6D,E). The qRT-PCR experimental data proved that YTHDC1 was positively correlated with hsa_circ_0058493 in HCC (Figure 6F). The location of hsa_circ_0058493 was detected by nuclear-cytoplasmic separation experiments which certified that U6 was present in the nucleus, 18S and hsa_circ_0058493 were present in the cytoplasm (Figure 6G). Next, we constructed a YTHDC1 knockdown plasmid and verified the knockdown efficiency of YTHDC1 (Figure 6H). When the YTHDC1 plasmid was knocked out, we found that hsa_circ_0058493 was transported from the cytoplasm to the nucleus (Figure 6I). Taken together, hsa_circ_0058493 is transported from the nucleus to the cytoplasm in a m6A-dependent manner by binding to YTHDC1.

### Overexpression of Hsa_circ_0058493 Rescued the Carcinogenic Effect of YTH Domain-Containing Protein 1 Knockdown in Hepatocellular Carcinoma

**FIGURE 7:**
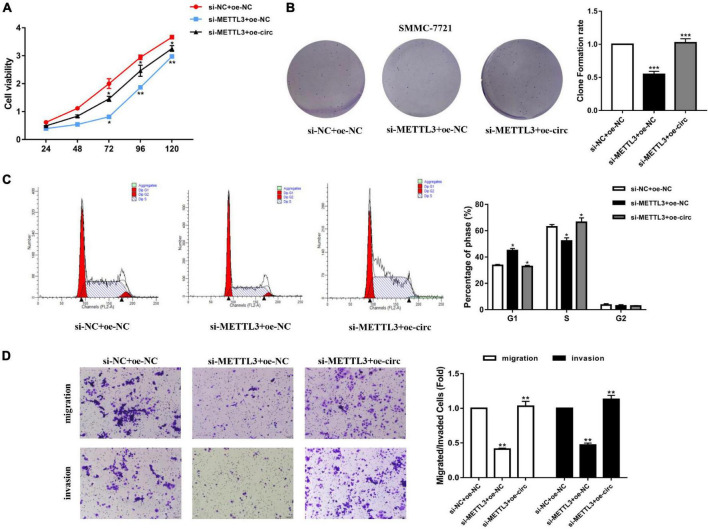
**(A)** A CCK-8 assay was tested to determine the proliferation of HCC cells with si-NC + oe-NC, si-METTL3 + oe-NC, and si-METTL3 + oe-circ. **(B)** A cloning experiment was used to detected cell proliferation ability of HCC cells with si-NC + oe-NC, si-METTL3 + oe-NC, and si-METTL3 + oe-circ. Colony formation rates are shown by histograms. **(C)** The cell cycle of HCC cells after transfection with si-NC + oe-NC, si-METTL3 + oe-NC, and si-METTL3 + oe-circ was determined by flow cytometry. As evident in the histogram, more cells arrested in the G1 phase were observed in si-METTL3 + oe-NC compared with si-NC + oe-NC. After cotransfection of si-METTL3 + oe-circ, the number of cells arrested in G1 phase decreased again compared with si-METTL3 + oe-NC. The triangle symbols are for discrimination of G1 vs. S and S vs. G2. **(D)** The invasion and migration abilities of HCC cells after transfection with si-NC + oe-NC, si-METTL3 + oe-NC, and si-METTL3 + oe-circ were assessed by Transwell assay. Histograms were used to show the number of invaded and migrated cells. *P < 0.05, **P < 0.01, ***P < 0.001 vs. control group.

After knocking down YTHDC1 (si-YTHDC1) and overexpressing hsa_circ_0058493 (oe-circ) in the SMMC-7721 cell line, the cell proliferation, migration and invasion abilities were determined. The CCK-8 experiment and the colony formation experiment proved that the cell proliferation ability decreased after knocking down YTHDC1. However, the proliferation ability was rescued after overexpression of hsa_circ_0058493 (Figures 7A,B). Afterward, flow cytometry analysis displayed that cell cycle arrest increased and cell growth ability decreased after transfection of the knockdown plasmid of YTHDC1 (si-YTHDC1), but transfection of the overexpression plasmid of hsa_circ_0058493 (oe-circ) rescued cell cycle arrest and promoted cell growth ability (Figure 7C). Similarly, the Transwell assay confirmed that after the knockdown plasmid of YTHDC1 (si-YTHDC1) was transfected into the SMMC-7721 cell line, the cell migration and invasion ability decreased which then was restored when the overexpression plasmid of hsa_circ_0058493 (oe-circ) was transfected, (Figure 7D). In summary, hsa_circ_0058493 promoted the growth and metastasis of HCC cells by binding to YTHDC1.

## Discussion

Hepatocellular carcinoma is a malignant tumor that occurs in the liver which has high mortality (Wang et al., 2020a). As one of malignant tumors, the pathogenesis of HCC, as well as its early diagnosis, treatment and prognosis in the clinic, has been of great interest (Wang et al., 2020b). In addition to surgical resection in HCC, alpha fetoprotein (AFP) is a currently known HCC diagnostic biomarker and widely used in the clinic (Wang and Wei, 2020; Zheng et al., 2020). However, early diagnosis by AFP is not available for all patients, and AFP is no longer recommended as a tool for HCC surveillance and diagnosis in recent HCC guidelines. Since the early diagnosis rate of HCC has not been satisfactory, the therapeutic effect of HCC has been poor (Wang and Zhang, 2020). Therefore, further analysis of the pathogenesis of HCC and search for new diagnostic- and prognostic-related markers has some significance for the treatment of HCC (Ozgor and Otan, 2020).

As a novel non-coding RNA that was first discovered many years ago, circRNA has a closed loop structure and is chiefly situated in the cytoplasm or existed in exosomes (Li et al., 2015). Most circRNAs are circularized from exons, or some have lasso structures circularized from introns (Xiao et al., 2020). Compared with linear RNA, circRNA is not affected by RNA exonuclease. Thus, circRNAs have more stable expressions and are not easily degraded (Verduci et al., 2019). With the advancement of contemporary sequencing technology, most circRNAs have been found in most eukaryotes. Recent studies have shown that circRNA is abnormally expressed in different diseases and plays a regulatory role in tumor development (Kong et al., 2020). For example, circRNA_0000285, with higher expression in cervical cancer (CC) than normal tissues, may promote the development of CC through FUS (Chen et al., 2019b). CircRNA_100876 expression was elevated in breast cancer (BC) and promoted the proliferation ability of BC cells (Yang et al., 2019). Hsa_circ_100395, with decreased expression in lung cancer tissues, inhibited the progression of lung cancer (Chen et al., 2018). With respect to our current study, we identified the cyclic structure of hsa_circ_0058493 and discovered that the expression of hsa_circ_0058493 was prominently upregulated in HCC. Afterward, survival curve analysis showed that hsa_circ_0058493 is a potential target for HCC. Hence, the promise of circRNAs for HCC treatment has been confirmed by numerous studies.

In this study, we investigated a new circular RNA, hsa_circ_0058493, and identified its circular structure. Subsequently, we conducted functional experiments to discuss the impact of hsa_circ_0058493 in the development of HCC. Some *in vivo* experiments verified that hsa_circ_0058493 can promote the growth and metastasis of HCC cells. In addition, hsa_circ_0058493 can also inhibit cell apoptosis. Similarly, *in vitro* nude mouse tumor formation experiments also proved that hsa_circ_0058493 can promote the growth of HCC tumors.

There are more than one hundred modifications of RNA, and N6-methyladenosine (m6A) modification is a common post-transcriptional modification of mRNA in mammals (Wang et al., 2020c). m6A methylation modification can affect many functions of mRNA, such as stability, splicing, export, translation and decay (Lee et al., 2020). Moreover, numerous documents have shown that m6A modification plays a crucial part in most cancers and that m6A modification exists in most non-coding RNAs (Huang et al., 2020).

**FIGURE 8:**
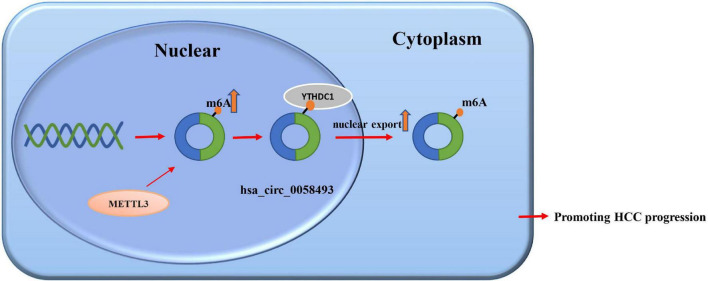
The regulatory mechanism of hsa_circ_0058493 in HCC.

Methyltransferase-like 3 is called the m6A methyltransferase “writer” and has been confirmed to be upregulated in HCC tissues. YTHDC1 possesses a YTH domain that has been generally accepted as a nuclear reader protein and preferentially binds m6A (Xu et al., 2014). In the present research, we discovered that METTL3 catalyzes m6A modification of hsa_circ_0058493, and the methylation site of hsa_circ_0058493 could bind to YTHDC1 and export it from the nucleus to the cytoplasm in a m6A-dependent manner, ultimately promoting HCC progression (Figure 8).

## Conclusion

We experimentally demonstrated that hsa_circ_0058493 was a meaningful oncogenic circRNA and may be a biomarker of HCC. Hsa_circ_0058493 played effects in HCC progression by promoting HCC cell growth and metastasis through the m6A-hsa_circ_0058493-YTHDC1 axis. The above results suggest that hsa_circ_0058493 may become a promising target for HCC and provide a strategy for HCC treatment.

## Data Availability Statement

The raw data supporting the conclusions of this article will be made available by the authors, without undue reservation.

## Ethics Statement

The studies involving human participants were reviewed and approved by the Ethics Committee of the Affiliated Hospital of Nantong University. The patients/participants provided their written informed consent to participate in this study. The animal study was reviewed and approved by the Ethics Committee of the Affiliated Hospital of Nantong University.

## Author Contributions

LC and FW conceived and designed the research. AW, YH, YX, XW, and AC performed the experiments. JX and RL analyzed the data. AW, YH, and YX wrote the manuscript. All authors reviewed and approved this version of manuscript.

## Conflict of Interest

The authors declare that the research was conducted in the absence of any commercial or financial relationships that could be construed as a potential conflict of interest.

## Publisher’s Note

All claims expressed in this article are solely those of the authors and do not necessarily represent those of their affiliated organizations, or those of the publisher, the editors and the reviewers. Any product that may be evaluated in this article, or claim that may be made by its manufacturer, is not guaranteed or endorsed by the publisher.
